# Effect of Imidazolium-Based Silver Nanoparticles on Root Dentin Roughness in Comparison with Three Common Root Canal Irrigants

**DOI:** 10.22037/iej.2017.17

**Published:** 2017

**Authors:** Melika Farshad, Abbas Abbaszadegan, Yasamin Ghahramani, Akram Jamshidzadeh

**Affiliations:** a *Student Research Committee, Dental School, Shiraz University of Medical Sciences, Shiraz, Iran; *; b *Department of Endodontics, Dental School, Shiraz University of Medical Sciences, Shiraz, Iran; *; c *Pharmaceutical Sciences Research Center, Shiraz University of Medical Sciences, Shiraz, Iran*

**Keywords:** Dentin Roughness, Imidazolium, Root Canal Irrigant, Silver Nanoparticle

## Abstract

**Introduction:**

The aim of this study was to evaluate the effect of a nanosilver-based irrigant on dentin roughness in comparison with three commonly used root canal irrigation solutions.

**Methods and Materials:**

Three common irrigants including 5.25% sodium hypochlorite (NaOCl), 17% ethylenediaminetetraacetic acid (EDTA) and 2% chlorhexidine (CHX) and also an imidazolium-based silver nanoparticle solution (ImSNP) (5.7×10 ^-8 ^mol/L), were used. Distilled water was used as control. Roots of 25 human anterior teeth were sectioned longitudinally to obtain 50 dentin samples. Roughness values were evaluated by atomic force microscopy analysis on 5 groups (*n*=10) after each group was treated in one of the tested irrigant solutions for 10 min. Values were statistically analyzed by One-way analysis of variance, followed by a post hoc Tukey’s test for pair-wise comparison.

**Results:**

Dentin roughness significantly increased from 95.82 nm (control) to 136.02 nm, 187.07 nm, 142.29 nm and 150.92 nm with NaOCl, CHX, ImSNP and EDTA, respectively. CHX demonstrated a significantly higher roughness value compared to the other tested irrigants while no significant differences were seen in NaOCl, ImSNP and EDTA groups (*P*>0.242).

**Conclusion:**

ImSNP affected the physicochemical properties of dentin and raised its surface roughness; thus, this irrigant could impact bacterial and restorative material adhesion to root canal dentin walls.

## Introduction

The antibacterial irrigation solutions express greater effectiveness in bacteria elimination [1]. Sodium hypochlorite (NaOCl) and sequential combination of NaOCl and ethylenediaminetetraacetic acid (EDTA) have been broadly used in root canal therapy for many years due to their antimicrobial characteristics, tissue solubility, affordability and ability to remove smear layer [2, 3]. Chlorhexidine (CHX) is another commonly used irrigation solution with an exemplary antimicrobial property against endodontic microorganisms including* Enterococcus faecalis *[2]*.*

As of yet, there has been no endodontic irrigant that has all the desired properties and therefore, the search for new irrigants is continuing. Undoubtedly nanosilver ions and silver nanoparticle-based (SNP) compounds are extremely toxic for microorganisms including 16 major species of bacteria while many studies have suggested that SNPs are compatible to human cells and they might impact human health only in high concentrations [4-6]. This characteristic of SNP makes it a suitable antibacterial choice in the medical field especially as a promising root canal disinfectant in dentistry [7, 8].

Mechanical debridement of infected root canals fails to fully remove debris from dentinal walls [9]. In order to chemically eliminate intracanal microorganisms, one or more intracanal irrigants may be used [1, 2]. While removing the smear layer, irrigation materials create a relative softening of the dentinal walls, which in turn facilitate the preparation of root canals [10]. On the other hand, decrease in the microhardness can affect the adhesion and sealing ability of the sealers to the root dentine walls [11]. Dentin surface roughness is an essential factor which might influence the bacterial and restorative material adhesion to root canal walls as well [12, 13]. Irrigation solutions can change this physicochemical property of dentin walls [12, 14]. Previous studies have revealed that two common endodontic irrigants, NaOCl and EDTA, increase dentin roughness [14-16]. Roughness can be measured through several methods. Researches using atomic force microscopy (AFM) have been conducted in numerous fields of dentistry [17-20]; however, only few reports are on application of this technique to measure dentin roughness values [13, 14].

Recently a new positively charged SNP irrigant coated by imidazole (ImSNP) has been introduced in the literature. This irrigant has more antimicrobial activity in very lower concentrations in comparison with CHX and NaOCl. Furthermore, dentin could not inhibit this irrigant and NaOCl at any concentrations after 24 h [7]. However, there is no report on the influence of this new irrigant on physicochemical properties of dentin.

The aim of this *in vitro* study is to evaluate the effect of ImSNP on dentin roughness in comparison with 5.25% NaOCl, 2% CHX and 17% EDTA using AFM analysis.

## Materials and Methods

The test irrigants included 17% EDTA (Sigma-aldrich corporation, St Louis, MO, USA), 2% CHX (Sigma-aldrich corporation, St Louis, MO, USA), 5.25% NaOCl (Sigma-aldrich corporation, St Louis, MO, USA), ImSNP(synthesized according to the protocol suggested by Abbaszadegan *et al. *[7] at 5.7×10 ^-8 ^mol/L), and fresh distilled water as control. In this study, roots of 25 human caries free permanent anterior teeth were cut-off from cementoenamel junction and then sectioned longitudinally under water to gain 50 dentin slices. In order to remove surface scratches samples were polished using 600-, 800- and 1200-grit polishing papers and were finally ultrasonicated in deionized water. Fifty dentin samples were randomly divided into 5 groups using simple randomization technique (*n*=10). 

Each group was treated with one of the tested (NaOCl, CHX, EDTA, ImSNP and deionized water) irrigants for 10 min. Samples were then rinsed with fresh deionized distilled water just prior to the measurements. Roughness (R) was evaluated by contact mode of AFM (Veeco, Santa Barbara, CA, USA) using V614r1 software (Veeco, Santa Barbara, CA, USA). Five separate 10×10 μm^2^ regions from different parts of radicular dentin in coronal, middle and apical portion of each root sample were selected and viewed. The R-values was measured and the averaged out to yield a single R-values for each dentin sample. 

R-values were statistically analyzed by one-way analysis of variance, followed by a post hoc Tukey’s test for pair-wise comparison. Statistical significance was set at 0.0.

## Results

**Figure 1:**
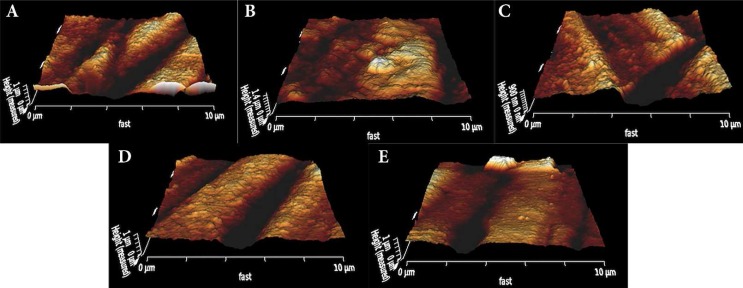
A) AFM image for typical dentin surfaces after treatment with NaOCl 5.25%, B) AFM image for typical dentin surfaces after treatment with CHX 2%, C) AFM image for typical dentin surfaces after treatment with ImSNP 5.7×10 ^-8^mol/L irrigant, D) AFM image for typical dentin surfaces after treatment with EDTA 17%, E) AFM image for typical dentin surfaces after treatment with distilled water

Table 1 describes the means and standard deviations of R-values. R-values significantly increased in all tested groups in comparison with control group (95.82 nm), (*P*<0.032). R-values of dentin samples treated with ImSNP (142.29 nm) was significantly lower than those treated with 2% CHX (187.07 nm); however, when it was compared to 5.25% NaOCl (136.02 nm) and 17% EDTA (150.92 nm) no significant differences were observed (*P*>0.242). Figures 1 to 5 demonstrate the AFM image samples for each group.

## Discussion

This study investigated the effect of a nano-silver irrigant (ImSNP) on dentin roughness using AFM on contact mode in comparison with three commonly used root canal irrigants (NaOCl, CHX, EDTA). All irrigants used in this study significantly increased the surface roughness of dentin samples. In our study, roughness of samples treated with ImSNP (142.29 nm) were significantly lower than those treated with 2% CHX (187.07 nm) while it was similar to those treated with 5.25% NaOCl (136.02 nm) and 17% EDTA (150.92 nm). To date there is no report on the effect of nanosilver solutions on surface roughness of dentin. The nature of an ionic liquid (imidazole) used as a stabilizer and also coating agent in the synthesis of ImSNP may explain the level of roughness induced by this solution. Furthermore, the dissimilarity in charge distribution on the cationic part of this molecule and dentin surface might also be another factor influencing dentin surface.

**Table 1:** Mean (SD) of roughness values of dentin samples for different irrigants (*n*=10) (Different letters in the column indicate a statistically significant difference

**Irrigant**	**Roughness values (Ra)**
**NaOCl 5.25%**	136.02 (25.81)^a^
**CHX 2%**	187.07 (24.02)^b^
**Ag NP ** **5.7×10 ** ^-8^ **mol/L**	142.29 (29.58)^a^
**EDTA 17%**	150.92 (25.20)^a^
**Distilled water**	95.82 (28.35)^c^

It is notable that surface roughness can be measured using various tools such as AFM, computerized roughness tester, and stylus profilometer. AFM was selected since it is able to characterize surfaces at extremely high resolution. A sharp probe is put into close proximity with the samples. Probe and samples are then moved relative to each other in a raster pattern, and a quantity is measured in a serial fashion at discreet locations [21]. Moreover, AFM is a software driven microscope which can work both in air and liquid and can report surface roughness value quantitatively. This microscope also provides very accurate three-dimensional images of surface topography. 

The obtained results regarding 5.25% NaOCl and 17% EDTA were similar to those reported by Hu *et al. *[14], who employed similar method as this study. Our findings were also compatible with the results by Ari *et al. *[16], who measured the surface roughness of dentin using computerized roughness tester. Chelating and smear layer removal property of EDTA which results in patency of dentinal tubules can describe how this irrigant increases dentin roughness [22]. Moreover, the effect of NaOCl on dentin roughness might be a result of collagen dissolving characteristic of this material [23, 24]. To the best of our knowledge, there is no report on the effect of 2% CHX on dentin roughness. We found that 2% CHX resulted in significantly, higher roughness compared to the other irrigants experimented. In previous studies by Leonardo *et al. *[25], and White *et al. *[26], it was suggested that CHX can adsorb dentin bonding agents within dentinal tubules. Hence, this mechanism can be an explanation for the effect of CHX on raising dentin roughness. Besides, previous investigations have revealed that premedication of dentin with CHX can significantly enhance the bonding of resin-based materials to root canal dentin [27-30].

As stated, the level of roughness can affect the bacterial adhesion to dentin. According to Kishen *et al. *[31], adhesion of *Enterococcus* *faecalis* increased in dentin samples treated with both NaOCl and EDTA, but not in samples treated with NaOCl alone. This indicates that bacterial adhesion is not only related to surface properties of dentinal wall, but it might be related to the type of bacteria and the irrigants used during the cleaning and shaping of the canal.

## Conclusion

Under the experimental condition of this study, we found that the newly introduced ImSNP could increase dentin roughness similar to the other commonly used irrigants and this can promote the adhesion of dentin to both restorative materials and microorganisms. Further investigations are required on the other properties of this material on physicochemical properties of dentin to justify its clinical application as a root canal irrigant.
